# Representing a Heterogeneous Pharmaceutical Knowledge-Graph with Textual Information

**DOI:** 10.3389/frma.2021.670206

**Published:** 2021-07-01

**Authors:** Masaki Asada, Nallappan Gunasekaran, Makoto Miwa, Yutaka Sasaki

**Affiliations:** Computational Intelligence Laboratory, Toyota Technological Institute, Nagoya, Japan

**Keywords:** drug database, knowledge graph embedding, knowledge graph completion, textual information, heterogeneous networks

## Abstract

We deal with a heterogeneous pharmaceutical knowledge-graph containing textual information built from several databases. The knowledge graph is a heterogeneous graph that includes a wide variety of concepts and attributes, some of which are provided in the form of textual pieces of information which have not been targeted in the conventional graph completion tasks. To investigate the utility of textual information for knowledge graph completion, we generate embeddings from textual descriptions given to heterogeneous items, such as drugs and proteins, while learning knowledge graph embeddings. We evaluate the obtained graph embeddings on the link prediction task for knowledge graph completion, which can be used for drug discovery and repurposing. We also compare the results with existing methods and discuss the utility of the textual information.

## 1 Introduction

Knowledge graphs (KGs) have attracted great attention from both academia and industry as a means of representing structured human knowledge. A wide variety of KGs have been proposed such as freebase (Bollacker et al., 2008), YAGO (Suchanek et al., 2007), and WordNet (Miller, 1995). A KG is a structured representation of facts that consists of entities, relations, and semantic descriptions. Entities are real-world objects and abstract concepts, relations represent relationships between entities, and semantic descriptions of entities, and these relationships include types and properties that have well-defined meanings. The KG usually consists of a set of triples {(h,r,t)}, where *h, r*, and *t* represent the head entity, relationship, and tail entity, respectively.

Recently, obtaining representation of KG elements in a dense vector space has attracted a lot of research attention. We have witnessed major advances in the KG expression learning model, which expresses entities and relationships as elements of a continuous vector space. The vector space embedding of all the elements in KGs have received considerable attention because it is used to create a statistical model of the whole KGs, i.e., to easily calculate the semantic distance between all elements and to predict the probabilities of possible relational events (i.e., edges) on the graph. Such models can be used to infer new knowledge from known facts (i.e., link prediction), to clarify entities (i.e., entity resolution), to classify triples (i.e., triple classification), and to answer the probability question and answering (Bordes et al., 2011; Nickel et al., 2011; Socher et al., 2013; Nickel et al., 2016). They can enhance knowledge learning capabilities from the perspectives of knowledge reasoning, knowledge fusion, and knowledge completion (Lin et al., 2016; Xie et al., 2016; Pham and Le, 2018; Ji et al., 2020).

Applications of the KG are often severely affected by data sparseness; however, a typical large-scale KG is usually far from perfect. The task of completing the KG aims to enrich the KGs with new facts. Many graph-based methods have been proposed to find new facts between entities based on the network structure of KG (Lao et al., 2011). Much effort has also been put into extracting relevant facts from plain text (Zeng et al., 2014). However, these approaches do not utilize KG information. Neural-based knowledge representations have been proposed to encode both entities and relationships in a low-dimensional space where new facts can be found in (Bordes et al., 2013; Lin et al., 2015). While traditional methods often deal with KGs without node types, in many real world data, entities have different semantic types. Recent methods deal with heterogeneous knowledge graphs with different types of nodes (Schlichtkrull et al., 2018; Wang et al., 2019). More importantly, neural models can be used to perform learning of text and knowledge within a unified semantic space to more accurately complete the KG (Han et al., 2016).

Representing text information in a vector space has also progressed rapidly. BERT (Bidirectional Encoder Representation from Transformers) (Devlin et al., 2019) is a pre-training model for NLP developed by researchers at Google AI Language. By providing the state-of-the-art findings in a wide range of NLP problems, including question answering and natural language inference, it has created a stir in the artificial intelligence community. The main technological innovation of BERT is to apply Transformer’s bidirectional modelling using self-attention for language modelling. Language models have traditionally only been able to read text input sequentially, either left-to-right or right-to-left, and they could not do both at the same time. BERT is distinct because it is built to read all together in both directions. This capability is recognized as bidirectionality, allowed by the invention of Transformers. Its purpose is to create a model of the language by pre-training the model on a large-scale text data. This gives it exceptional precision and efficiency on smaller data sets, addressing a major problem in the NLP and an highly expressive way to represent texts.

Nowadays, there has been a lot of interest in jointly learning KG and embedding textual information. However, traditional KG models based on representation learning only use structural information embedded in a particular KG. Plain text textual information, on the other hand, provides a wealth of semantic and contextual information that can contribute to the clarity and completion of entity representations and relationship representation of a given KG. Therefore, textual information can be seen as an effective supplement to the completed task of the KG. In order to explore the informative semantic signals of plain text, there has recently been a great deal of interest in learning together the embeddings of KG and text information in (Toutanova et al., 2015). Moreover, the researchers provided a text-enhanced KG representation model that utilized textual information to enhance the knowledge representations (Wang et al., 2020).

The pharmaceutical field is a good target of applying such text-enhanced KG models. Side effects impose a financial burden on the health-care system due to additional hospitalization, morbidity, mortality, and the cost of health care utilization. The occasional drug-drug interactions (DDIs) caused by the co-prescribing of a drug with another drug can cause undesired effects other than its major pharmacological effects (Abubakar et al., 2015). The significant number of drug side effects (about 3–26%) that lead to hospitalization are due to unintended DDIs in (Dechanont et al., 2014). Patient groups, such as the elderly and cancer patients are more likely to take multiple medications at the same time, which is increasing the risk of DDIs (Riechelmann et al., 2007; Doubova et al., 2007). Current approaches to identifying DDIs, such as safety investigations during drug development and post-approval marketing monitoring, provide an important opportunity to identify potential security issues, but cannot provide complete to all possible DDIs in (Percha and Altman, 2013). Therefore, drug discovery researchers and health professionals may not be fully aware of dangerous DDIs. Predicting potential DDIs can help reduce unexpected drug reactions and drug development costs and improve the drug design process. Therefore, there is a clear need for automated methods for predicting DDIs. Several efforts have been made to automatically collect DDI information from biomedical literature using text mining tools in (Zhao et al., 2016; Simon et al., 2019; Asada et al., 2020; Wu et al., 2020). They are not enough to predict potential DDIs and we need a way to predict such potential DDIs.

Methods for computational drug repurposing and drug discovery include chemoinformatics-based methods, network-based methods, and data- or text-mining-based methods. Some approaches to drug repurposing rely on data- and text-mining and are based on identification of patterns in databases or natural language text to predict novel associations between drugs and targets or drugs and diseases (Andronis et al., 2011; Sheikhalishahi et al., 2019; Wang et al., 2021). Since drug interactions are widely published in publications, medical literature is the best source for detecting them. Information Extraction (IE) can be very useful in the pharmaceutical industry, allowing for the detection and extraction of specific data on DDIs and offering a fun way for health care practitioners to spend less time reading the literature. The aim of this work is to create a common structure for evaluating knowledge extraction techniques used in biomedical texts for recognizing pharmacological substances and detecting DDIs, which motivates our present study.

As the knowledge graph grows, many of the world’s leading researchers have succeeded in obtaining information from vast medical databases and creating the largest heterogeneous graphs that reflect the clinical realities of drugs and diseases. For example, the DrugBank (Law et al., 2014) is a rich source of medical information. This includes a wide range of organizations (drugs, pharmaceutical targets, chemistry, etc.) and relationships (such as enzyme pathways, DDIs, etc.). Recently, researchers designed for speed, efficiency, and robustness through the use of a graph database of an ICD-9 ontology (Schriml et al., 2012) and refers to the knowledge base of human disease and can be used to classify a patient’s diagnosis. Using a well-structured clinical knowledge graph with an EMR-based clinical prescription system, the restructured system provides the right medications for specialized patients, as well as alerts of potential side effects and serious DDIs. To the best of authors knowledge, the heterogeneous pharmaceutical knowledge-graph with textual information have not been studied yet.

Based on the above motivation, this paper investigates a heterogeneous pharmaceutical knowledge-graph containing textual information constructed from several databases. We construct the heterogeneous entity items consisting of drug, protein, category, pathway, and Anatomical Therapeutic Chemical (ATC) code, and relations among them, which include category, ATC, pathway, interact, target, enzyme, carrier, and transporter. We compare three methods to incorporate text information in KG embedding training with representing text with BERT. We evaluate the resulting node and edge embeddings by the link prediction task and verify the usefulness of using text information in KG embedding training. The study of KG completion is roughly divided into two types: a study in which the link prediction task is performed by using score functions such as TransE (Bordes et al., 2013), DistMult (Yang et al., 2014), and a study in which Graph Convolutional Networks (Kipf and Welling, 2017), etc, are applied to the whole KG, and the node classification task is performed. In this study, we focus on the link prediction task and investigate the usefulness of text information in scoring function-based link prediction tasks.

Our contributions are summarized as follows:

• We propose a heterogeneous knowledge graph with textual information (called *PharmaHKG*) in the drug domain. This can be used to develop and evaluate heterogeneous knowledge embedding methods.
• We propose three methods to incorporate text information into KG embedding models.
• We evaluate and compare the combinations of four KG embeddings models and three methods to integrate text information on the link prediction task in the proposed knowledge graph, and we show there is no single method that can perform best for different relations and the best combination depends on the relation type.

## 2 Materials and Methods

In this section, we first introduce a heterogeneous pharmaceutical knowledge graph PharmaHKG that is constructed in this paper. We then explain the definition of KG and the learning method of embeddings in KG. We finally explain our proposed method that effectively uses text information for KG representation learning.

### 2.1 Heterogeneous Pharmaceutical Knowledge Graph with Textual Information

**FIGURE 1:**
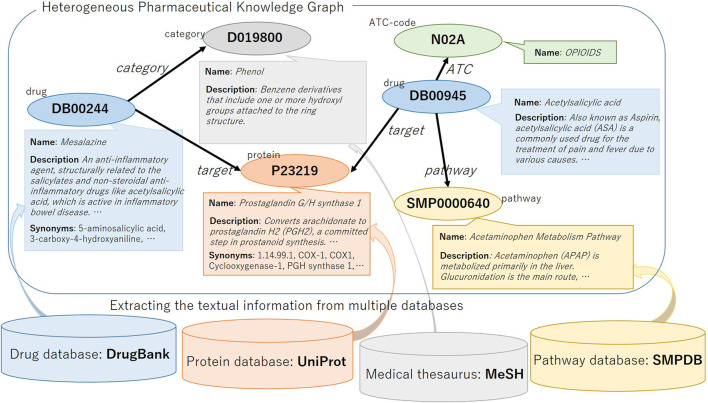
Illustration of the heterogeneous pharmaceutical knowledge graph.

We construct a heterogeneous pharmaceutical knowledge graph with textual information from DrugBank (Wishart et al., 2017) and its relating data sources. DrugBank is one of the rich drug databases. It contains several different types of nodes, which can be a good source for a heterogeneous knowledge graph. The nodes are related to several textual information in DrugBank and their linked entries in several other data sources such as UniProtKG (Consortium, 2018), Small Molecule Pathway Database (SMPDB) (Jewison et al., 2014) and medical thesaurus Medical Subject Headings (MeSH). The existence of such textual information fits our objective to evaluate the utility of textual information in knowledge graph representation. We illustrate the KG and the related data sources in Figure 1. In this section, we first explain the nodes and relations in the KG and then explain the textual information.

#### 2.1.1 Heterogeneous Pharmaceutical Knowledge Graph

We construct a KG consisting of five different types of heterogeneous items, i.e., drug, protein, pathway, category, and ATC code, from different databases and thesaurus. The statistics of the number of nodes is shown in Table 1.

• **Drug**: We extract information of drugs from DrugBank (Wishart et al., 2017). More than 10,000 drugs are registered in DrugBank, and various types of information such as drug names, descriptions, molecular structures and experimental properties are registered.
• **Protein**: We extract the information of proteins from UniProtKG (Consortium, 2018). UniProtKG consists of Swiss-Prot which is manually annotated and reviewed and TrEMBL which is automatically annotated and not reviewed, and we use Swiss-Prot knowledgebase.
• **Pathway**: We extract information of pathways from Small Molecule Pathway Database (SMPDB) (Jewison et al., 2014). SMPDB is an interactive, visual database containing more than 30,000 small molecule pathway found in humans.
• **Category**: We extract information of drug categories from medical thesaurus Medical Subject Headings (MeSH) (Lipscomb, 2000). Each drug recorded in DrugBank has several hypernymy categorical classes and these classes have MeSH term ID. As an example, a drug *Morphine* has categories such as *Alkaloids* (MeSH ID:D000470), *Anesthetics* (MeSH ID:D018681), and the detailed information can be obtained by referring to MeSH.
• **ATC**: Anatomical Therapeutic Chemical (ATC) classification system also has categorical information of drugs. In the ATC classification system, drugs are divided into different groups according to the organ or system on which they act and their therapeutic, pharmacological, and chemical properties. Drugs are classified in groups at five different levels. The drugs are divided into fourteen main groups (first level), with pharmacological or therapeutic subgroups (second level). The third and fourth levels are chemical/pharmacological/theraperutic subgroups and the fifth level is the chemical substance. For example, a drug “Metformin” is classified into “A: Alimentary tract and metabolism” (first level), “A10: Drugs used in diabetes” (second level), “A10B: Blood glucose lowering drugs, excl. insulins” (third level), “A10BA: Biguanides” (fourth level) and “A10BA02: metformin” (fifth level).

**TABLE 1:** Statistics of heterogeneous pharmaceutical KG entities.

Entity type	#
Drug (DrugBank-ID)	11,516
Protein (Uniprot-ID)	5,339
Pathway (SMPDB-ID)	874
Category (MESH-ID)	2,166
ATC (ATC-code)	1,093
Total	20,988

Five different types of nodes are connected by the following eight types of relations: *category*, *ATC*, *pathway*, *interact*, *target*, *enzyme*, *carrier*, and *transporter*. The statistics of the KG edges for each relation type is shown in Table 2. We extract the relation triples from DrugBank.

**TABLE 2:** Statistics of heterogeneous pharmaceutical KG edges for each relation type.

Relation type	All	Train	Valid	Test
Category	60,459	54,419	3,020	3,020
ATC	16,341	14,711	815	815
Pathway	18,707	16,847	930	920
Interact	2,682,142	2,413,932	134,105	134,105
Target	18,467	16,627	920	920
Enzyme	5,206	4,686	260	260
Carrier	815	735	40	40
Transporter	3,093	2,793	150	150
Total	2,750,228	2,525,829	140,240	140,240

Drug nodes and MeSH categorical terms are linked by *category* relation.

• *category*: This relation type indicates the MeSH category of drugs. These relationship indicates that the drug is classified into the therapeutic category or the general category (anti-convulseant, antibacterial, etc.) defined by MeSH. These relationship are registered by the manual search of DrugBank developers. These relationship indicates that the drug is classified into the therapeutic category or the general category (anti-convulseant, antibacterial, etc.) defined by MeSH. These relationship are registered by the manual search of DrugBank developers.

Drug nodes and ATC classification system codes are linked by *ATC* relation. In order to incorporate hierarchical information into the KG, we link ATC codes to ATC codes by *ATChypernym* relation. ATC codes are linked to the next higher level codes with this relation. We create relational triples such as A10BA—ATChypernym—A10B, N02—ATChypernym—N by linking the ATC code of the next higher level. Since this relation is apparent from the surface strings of ATC codes, we do not consider this relation for link prediction.

• *ATC*: Drugs are linked to any level of ATC codes with this relation. In DrugBank database, drug elements may have one or more ATC-code elements, e.g., drug *Morphine* four ATC codes (A07DA52, N02AA51, N02AA01 and N02AG01), and each ATC-code element has child elements. All these child entities and the drug entity are connected by the *ATC* relation.

Drug nodes and protein nodes are also connected with pathways.

• *pathway*: This relation type indicates a drug or protein is included in a pathway. When the drug is involved in metabolic, disease, and biological pathways as identified by the SMPDB, the drug entity and the pathway entity is connected by the *pathway* relation. Also, when the enzyme protein is involved in the same pathways, the protein entity and the pathway entity are connected by the relation.

Drug nodes can be connected by a relation *interact*.

• *interact*: A triple of this relation type indicates that the drug pair has a DDI. When concomitant use of the pair of drugs will affect its activity or result in adverse effects, these two drug entities are connected by *interact* relation. These interactions may be synergistic or antagonistic depending on the physiological effects and mechanism of action of each drug.

Drug nodes and protein nodes can be linked by *target*, *enzyme*, *carrier*, or *transporter* relation.

• *target*: A protein, macromolecule, nucleic acid, or small molecule to which a given drug bids, resulting in an alteration of the normal function of the bound molecule and a desirable therapeutic effect. Drug targets are most commonly proteins such as enzymes, ion channels, and receptors.
• *enzyme*: A protein which catalyzes chemical reactions involving a given drug (substrate). Most drugs are metabolized by the Cytochrome P450 enzymes.
• *carrier*: A secreted protein which binds to drugs, carrying them to cell transporters, where they are moved into the cell. Drug carriers may be used in drug design to increase the effectiveness of drug delivery to the target sites of pharmacological actions.
• *transporter*: A membrane bound protein which shuttles ions, small molecules, or macromolecules across membranes, into cells or out of cells.

#### 2.1.2 Textual Information of Knowledge Graph

Here we explain the text information relating to each type of node.

• **Drug**: Drugs are assigned a unique DrugBank-id. We use various text information contained in the DrugBank xml file. “Name,” to heading of the drug and standard name of the drug as provided by the drug manufacturer, “Description,” which describes the general facts, composition and/or preparation of the drug, “Indication” is a description or common names of diseases that the drug is used to treat, “Pharmacodynamics” is a description of how the drug works at a clinical or physiological level, “Mechanism of Action” is a description of how the drug works or what it binds to at a molecular level, “Metabolism” is a mechanism by which or organ location where the drug is neutralized, and “Synonyms” indicates alternate drug names.
• **Protein**: Protein targets of drug actions, enzymes that are inhibited/induced or involved in metabolism, and carrier or transporter proteins involved in movement of the drug across biological membranes. Each of *targets*, *enzymes*, *carriers*, *transporters* have unique UniProt-id. We refer to UniProt-id and obtained the following types of textual information, functions, miscellaneous description, short name, alternative names, gene names.
• **Pathway**: We extract pathway relations from DrugBank database. Each pathway has a unique ID defined by The Small Molecule Pathway Database (SMPDB) (Jewison et al., 2014). “Name” and “Description” of the pathway are registered in SMPDB.
• **Category**: Drug categories are classified according the medical thesaurus MeSH. These textual information are registered in MeSH: “Name” is a definition word, “ScopeNote” is a term description, “Entry terms” is a synonym.
• **ATC**: Drugs are classified in a hierarchy with five different levels by WHO drug classification system (ATC) identifiers. Each level of ATC classification code has a name, which is defined as the international nonproprietary name (INN) or to the name of the ATC level. We use these names given to ATC codes as textual information.

### 2.2 Learning Knowledge Graph Embeddings

#### 2.2.1 Knowledge Graph Definition

We treat a heterogeneous knowledge graph (KG) as a directed graph whose nodes and edges have semantic types. The semantic types are assigned to different types of nodes (drug, protein, pathway, etc.) and relations (target, carrier, etc.) to represent detailed information of nodes and relations. A KG is defined as a directed graph G=(E,R,F), where the nodes *E* denotes the set of typed entities, *R* refers to the set of typed relations and *F* represents the set of facts (i.e., directed edges). The nodes are often called entities. The facts or directed edges are often called triplets and are represented as a (h,r,t) tuple, when *h* is the head entity, *t* is the tail entity and *r* is the relation from the head entity to the tail entity.

#### 2.2.2 Scoring Functions

The methods that represent KG by using embeddings of entities and relations can catch the structure information of the KG and provide structure-based embeddings. Entities and relations are directly represented as the real-valued vector, matrix or complex-valued vectors. Scoring function f(h,r,t) is defined on each triple (h,r,t) to access the validity of triples. Triples observed in the KG tend to have higher scores than those that have not been observed. We employ the following four scoring functions.

##### 2.2.2.1 TransE

TransE (Bordes et al., 2013) is a representative translational distance model that represents entities and relations as vectors in the same semantic space of dimension ${\mathbb{R}}^{d}$ where *d* is the dimension of the target space with reduced dimension. A fact in the source space is represented as a triplet (h,r,t). The relation ship is interpreted as a translation vector so that the embedded entities are connected by relation *r* have a short distance. In terms of vector computation, it could mean adding a head to a relation should we set the norm to 2, so the scoring function is computed as:

$$f\left(h,\;r,\;t\right)=-{\left|h+r-t\right|}_{2}\text{.}$$ (1)

##### 2.2.2.2 DistMult

DistMult (Yang et al., 2014) is a method that speeds up the RESCAL model (Nickel et al., 2011) by considering only symmetric relations and restricting *M*_*r*_ from a general asymmetric *r × r* matrix to a diagonal square matrix, thus reducing the number of parameters per relation to *O(d)*. DistMult scoring function is computed as:

$$f\left(h,r,t\right)=h^{T}\text{diag}\left(r\right)t=\sum_{i=0}^{d-1}{\left[h\right]}_{i}{\left[r\right]}_{i}{\left[t\right]}_{i}.$$ (2)

##### 2.2.2.3 ComplEx

ComplEx (Trouillon et al., 2016) uses complex vector operations to consider both symmetric and asymmetric relation. The scoring function for complex entity and relation vectors h, r, and $t\in {\mathbb{C}}^{d}$ is computed as:

$$f\left(h,r,t\right)=\text{Real}\left(h^{T}\text{diag}\left(r\right)t\right)\text{,}$$ (3)

where Real extracts real part of the complex vectors.

##### 2.2.2.4 SimplE

SimplE (Kazemi and Poole, 2018) considers two vectors $h,t\in {\mathbb{R}}^{d}$ as the head and tail embeddings for each entity and two vectors $v_{r},v_{r^{-1}}\in {\mathbb{R}}^{d}$ for each relation *r*. The similarity function of SimplE for a triple (h,r,t) is defined as:

$$f\left(h,r,t\right)=\frac{1}{2}\left(\langle h_{h},v_{r},t_{t}\rangle +\langle h_{t},v_{r^{-1}},t_{h}\rangle \right).$$ (4)

We chose the above four score functions because these are widely used and cover the standard ideas for scoring relational triples: distance-based, bilinear-based and complex number-based.

#### 2.2.3 Negative Sampling and Loss Functions

Generally, to train a KG embedding, the models apply a variation of negative sampling by corrupting triplets (h,r,t). They corrupt either *h*, or *t* by sampling from set of head or tail entities for heads and tails, respectively. The corrupted triples can be either of $\left(h^{\prime },r,t\right)$ or $\left(h,r,t^{\prime }\right)$, where $h^{\prime }$ and $t^{\prime }$ are the negative samples. We acknowledge that due to the incompleteness of the current KG, the unregistered and potentially positive relational triples can be negative examples: this problem is common to most studies that tackle with the link prediction task. To avoid easy negative samples and utilize the entity type information, we restricted the node types of negative samples depending on *r*. The logistic loss and the margin based pairwise ranking loss are commonly used for training. The logistic loss returns −1 for negative samples and +1 for the positive samples. $D^{+}$ and $D^{-}$ are negative and positive data, y=±1 is the label for positive and negative triples, and f(⋅) is the scoring function. Model parameters are trained by minimizing the negative log-likelihood of the logistic model with L2 regularization on the parameters Θ of the model;

$$L_{KG}\sum_{\left(h,r,t\right)\in D^{+}\cup D^{-}}\log\left(1+\text{exp}\left(y\times f\left(h,r,t\right)\right)\right)+\lambda {\left\|\Theta \right\|}_{2}^{2}.$$ (5)

The margin based pairwise ranking loss minimizes the rank for positive triples. Ranking loss is given by:

$$L_{KG}=\sum_{\left(h,r,t\right)\in D^{+}}\sum_{\left(h^{\prime },r^{\prime },t^{\prime }\right)\in D^{-}}\text{max}\left(0,\gamma -f\left(h,r,t\right)+f\left(h^{\prime },r^{\prime },t^{\prime }\right)\right)+\lambda {\left\|\text{Θ}\right\|}_{2}^{2}\text{.}$$ (6)

### 2.3 Utilizing Textual Information

**FIGURE 2:**
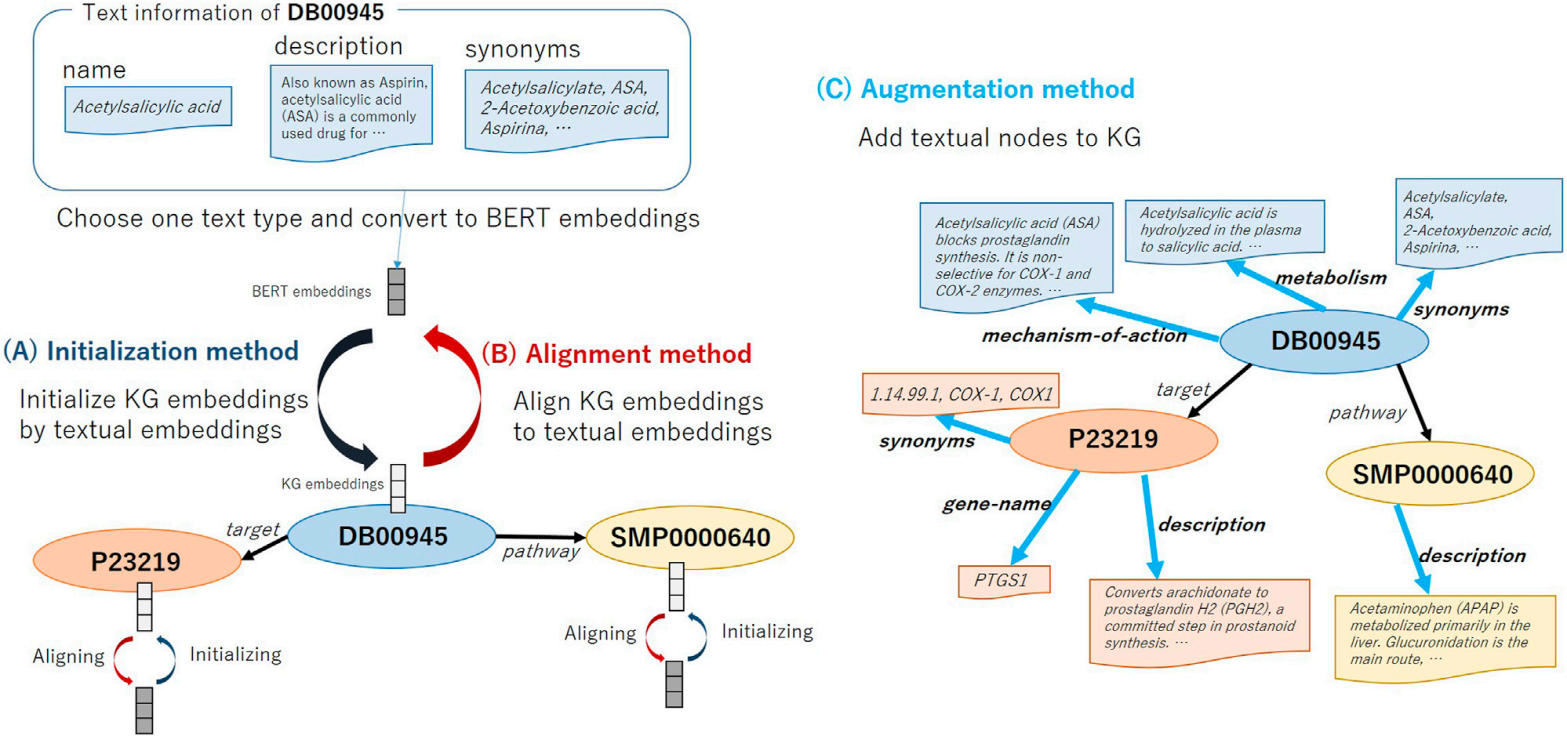
Overview of methods: **(A)** Initializing node embeddings (Initialization), **(B)** Aligning entity embeddings and textual embeddings (Alignment), and **(C)** Augmenting KG embeddings (Augmentation).

In this study, we verify the usefulness of using text information in KG embedding training by three methods explained below. Figure 2 shows the overview of the three methods that utilize text information for KG embedding representation. We employ Bidirectional encoder representation from transformer (BERT) (Devlin et al., 2019), which is an extremely high-performance contextual language representation model, in encoding text. BERT is pre-trained with the masked language model objective and next sentence prediction task objective on large unlabeled corpora, and fine-tuned BERT towards the target task achieved the state-of-the-art performance.

#### 2.3.1 Initializing Node Embeddings

Usually, the initial value of embedding for each node in KG is given randomly in the existing methods. As shown in Figure 2A, first, we select which type of text to use, e.g., drug nodes have text types such as Name, Description and Synonyms. We then take the selected text as the input of the text encoder model BERT and the <CLS> embeddings of the BERT as the initial value of the node embeddings. For the methods that use two embeddings for an entity, i.e., ComplEx (real and imaginary embeddings) and SimplE (head and tail embeddings), we initialize both vectors with the <CLS> embeddings. When multiple text items are registered (e.g., the drug Acetaminophen has multiple synonyms, “Acenol,” “APAP,” “Paracetamol”), we connect these terms with a comma and take it as an input for BERT. We call this the *Initialization* method. The motivation of the *Initialization* method is to help representing node embeddings by using the BERT embeddings that pre-trained on a large amount of biomedical literature. We aim to predict correct relational triples from textual information by BERT even if the structural information of the graph is insufficient.

#### 2.3.2 Aligning Entity Embeddings and Textual Embeddings

The aligning method aims to gradually project KG embeddings into textual embeddings space by adding the regularization term to loss function.

$$L_{a}=\lambda_{a}{\left\|V_{KG}-V_{text}\right\|}_{2}\text{,}$$ (7)

$$L=L_{KG}+L_{a}\text{,}$$ (8)

where $\lambda_{a}$ is a regularization coefficient of alignment, $V_{KG}$ and $V_{text}$ are vector lookup table matrices of KG and textual embeddings, respectively. Similarly to the initialization method, the textual embeddings are obtained from BERT and when there are two embeddings for an entity, we regularized both vectors. We call this the *Alignment* method. The motivation of the *Alignment* method is that as the updating the node representation progresses, the two spaces of the text embeddings and the graph structural embeddings are projected into the same space, and finally we obtain more suitable node representations.

#### 2.3.3 Augmenting Knowledge Graph Embeddings

In this method, as shown in Figure 2C, we augment the KG structure by adding relation triples based on the text information of the node. The node’s own embedding is initialized with textual embeddings of Name. The embedding value of linked nodes is initialized with the BERT output. Moreover, since ATC classification codes have a hierarchical structure as shown in Figure 2C, after extending the link from the drug node to create new categorical nodes, further linking is made between the categorical nodes. We construct a graph that can consider both text information and the hierarchical information. We call this the *Augmentation* method. The motivation of the *Augmentation* method is to consider multiple text information of one entity at once.

## 3 Results and Discussions

### 3.1 Experimental Settings

#### 3.1.1 Constructing Heterogeneous Knowledge Graph with Textual Information

We show the overview of constructing a heterogeneous KG with textual information in Figure 1. We downloaded four publicly available databases, DrugBank, UniProt, MeSH term descriptions and SMPDB, and first we processed DrugBank and extracted relations between the drug and other heterogeneous items. Here, the text information of each drug are also extracted and associated with the entity ID in the KG. Next, for entities other than drugs, we used the link ID of DrugBank to refer to other databases and associated the text information with entity ID in the KG. As a result, five types of entities (i.e., drug, protein, pathway, category, and ATC) are included in the constructed KG. Between entities, there are relation links: category, ATC, pathway, interact, target, enzyme, carrier, and transporter. The total number of relational triples is about 2.7 M, and as shown in Table 2, the number of drug-interact-drug triples is large and accounts for the majority of them. Note that only the relation drug-interact-drug is symmetric, and the other relations are asymmetric, that is, when there is a DrugA-interact-DrugB relation triple in the KG, there is also a DrugB-interact-DrugA triple.

#### 3.1.2 Encoding Text Information

We employed PubMedBERT (Gu et al., 2020) to encode textual information into fixed-length real-valued embeddings. PubMedBERT is a model that uses 21B words of PubMed corpus for pre-training, and it shows high performance in several NLP tasks in the biomedical domain. In this paper, we used texts such as names and descriptions as inputs for pre-trained PubMedBERT, and used the output <CLS> token embedding as a textual representation. We set the maximum length of the input subword to 512.

#### 3.1.3 Knowledge Graph Embedding Training Settings

We employed four knowledge graph embedding scoring functions as explained in Section 2.2.2. For each of the scoring functions, we applied three methods to train embedding using textual information; the initialization, aligning and the augmenting methods.

We show the ratio of nodes that have each textual information in Table 3. The node has the text information of Name in any database. In UniProt database, most proteins have Description and Synonyms texts information, and many categorical terms in MeSH also have Description and Synonyms. On the other hand, some drugs in DrugBank do not have some text information. In the Initialization method and Alignment method, one text type is selected and the embeddings of textual information are used.^1^ When the node does not have the text information, the text of Name is used instead.

**TABLE 3:** The percentage of nodes that have each type of text.

	Name	Description	Synonyms (%)
Drug	100	53.72	48.50
Protein	100	96.17	100
Category	100	94.01	91.42
ATC	100	—	—
Pathway	100	100	—

Nodes in all databases have a Name text information. While many proteins and categories have Description information and Synonyms information, the percentage of drugs that have these information is low.

Drugs and proteins have textual information that other nodes do not have, and their coverage is as follows: 32.61% drugs have Indication information, 24.60% drugs have Pharmacodynamics information, 18.40% drugs have Metabolism information, 30.52% drugs have Mechanism-of-action information and 96.05% of proteins have Gene-name. These text items are linked to the KG nodes in the Augmentation method, so the Augmentation method can utilize all text information.

We prepared the random initialization method without textual information (*No Text*) as the baseline. In this setting, embeddings of entities and relations are initialized with the random values drawn from a uniform distribution between $\pm \left(\gamma +\frac{\epsilon }{d}\right)$, where γ=12, ϵ=2 and *d* is a dimension of KG embeddings.

#### 3.1.4 Task Setting

We evaluated the node and edge embeddings by the link prediction task. Link prediction is a task to search an entity which probably constructs a new fact with another given entity and a specific relation. For KGs are always imperfect, link prediction aims to discover and add missing knowledge into it. With the existing relations and entity, candidate entities are selected to form a new fact. We replace the head or tail of the triples in the validation or test data set with other entities that have the same entity types and calculate the scores of all created negative triples in the KG. We sort the calculated positive triple score and the scores of all negative triples and evaluate the rank of the positive triple score. Mean reciprocal rank (MRR) is used as evaluation metric. When we create negative example triples, if there are correct triples that exist in the KG, we excluded such triples from the ranking. This evaluation setting has been adopted in many existing studies as a **filtered** setting (Bordes et al., 2013; Trouillon et al., 2016; Kazemi and Poole, 2018). In addition, similar to the negative sampling setting during training, given the relational edge label, the node types of head or tail are trivial, so we also excluded triples with inappropriate combinations of edge and node types.

We divided the extracted approximately 2.7 M relational triples into 90:5:5 as train, valid and test data sets. In the augmentation method, relational triples created from textual nodes are added to the train data set.

#### 3.1.5 Hyper-Parameter Settings

We tuned hyper-parameters by evaluating the MRR score on validation set for each model. We choose hyper-parameters with following values: regularization coefficient $\lambda \in \left\{10^{-3},10^{-6},10^{-9},10^{-12},0\right\}$, alignment regularization coefficient $\lambda_{a}\in \left\{10^{-3},10^{-6},10^{-9},10^{-12}\right\}$, initial learning rate $\alpha_{0}\in \left\{0.5,0.25,0.1,0.05,0.025,0.01\right\}$, For loss function, we adopted pair-wise hinge loss function for TransE and DistMult and logistic loss function for ComplEx and SimplE according to the setting of the original papers. The KG embedding dimension is set to 768 in order to match the dimension of the output of BERT embedding. For all models, we set the batch size for 4,096 and the number of epochs for 100.

#### 3.1.6 Implementation Details

We implemented all the models using the PyTorch library (Paszke et al., 2019), the DGL-KE library (Zheng et al., 2020) for knowledge graph embeddings, and the transformers library (Wolf et al., 2020) for BERT. We modified the original DGL-KE implementation in the following point. While DGL-KE samples negative examples from all combinations of entity pairs, our model excludes impossible negative instances by restricting the types of entities by the relations (e.g., a drug-interact-category triple is not created for negative samples) as explained in Section 2.2.3.

## 3.2 Results

Table 4 shows the comparison of link prediction MRR for each relation edge type, the macro-averaged MRR. While a micro-average MRR is calculated by directly calculating the MRR for all instances in the KG without considering the types, a macro-averaged MRR is calculated by first calculating the MRR for each type and then taking the average of the MRR scores. Since the constructed triples are highly imbalanced and the proportion of interact triples is large, models with high prediction performance of relation *interact* can result in high micro-averaged MRR. We report the macro-averaged MRR to avoid the effect of this imbalance. For each scoring function, we showed the comparison of performance between the models with and without text information.

**TABLE 4:** Comparison of MRR performance for each method.

	No text	Initialization			Alignment			Augmentation
	—	Name	Desc	Syn	Name	Desc	Syn	—
TransE								
Category	0.1978	0.2117	0.2120	0.2231	0.2136	0.2059	0.1913	**0.2239**
ATC	0.2929	0.2695	0.2571	0.2495	**0.3000**	0.2973	0.2872	0.2571
Pathway	**0.6741**	0.5674	0.5792	0.5793	0.6694	0.6711	0.6713	0.5473
Interact	**0.3109**	0.2867	0.2845	0.2843	0.3103	0.3106	0.3108	0.2644
Target	0.0802	0.0748	0.0811	0.0808	0.0808	0.0780	0.0821	**0.0889**
Enzyme	0.3262	0.3067	0.3314	0.3090	0.3474	0.3564	**0.3590**	0.2926
Carrier	**0.4155**	0.3078	0.2843	0.3679	0.4037	0.4044	0.4010	0.3459
Transporter	**0.3576**	0.3194	0.3104	0.3182	0.3444	0.3308	0.3391	0.2866
Avg. (macro)	0.3319	0.2930	0.2950	0.3015	**0.3337**	0.3318	0.3302	0.2883
DistMult								
Category	0.2539	**0.2876**	0.2797	0.2753	0.2679	0.2661	0.2586	0.2649
ATC	0.2428	0.2674	**0.2899**	0.2639	0.2612	0.2617	0.2698	0.2904
Pathway	**0.6792**	0.5424	0.5542	0.6002	0.6524	0.6711	0.6615	0.4997
Interact	0.7730	0.6338	0.5895	0.6302	0.7911	0.7868	**0.7990**	0.6113
Target	0.0738	0.0866	0.0864	0.0947	0.0778	0.0758	0.0734	**0.1049**
Enzyme	0.2501	0.2516	0.2358	**0.2847**	0.2247	0.2334	0.2140	0.2183
Carrier	0.2023	**0.2254**	0.1640	0.1622	0.1369	0.1311	0.1649	0.2134
transporter	0.2293	**0.2703**	0.1840	0.2190	0.1969	0.1939	0.1708	0.2062
Avg. (macro)	**0.3380**	0.3206	0.2979	0.3162	0.3261	0.3274	0.3265	0.3011
ComplEx								
Category	0.0905	0.3455	**0.3495**	0.3386	0.3302	0.0577	0.0611	0.3420
ATC	0.3326	0.3463	0.3623	0.3485	0.3271	0.3425	0.3407	**0.3652**
Pathway	0.6956	0.6856	0.7051	0.7157	0.7220	0.6963	**0.7323**	0.6820
Interact	**0.8678**	0.7632	0.7166	0.7802	0.8578	0.8189	0.8497	0.8230
Target	0.0496	0.1116	0.1093	0.1153	0.0859	0.0640	0.0740	**0.1243**
Enzyme	0.2103	0.2256	0.2512	**0.2538**	0.2245	0.1907	0.2097	0.2073
Carrier	0.1533	0.1557	0.1817	0.1423	0.0994	0.1750	0.1462	**0.1934**
transporter	0.1942	**0.3119**	0.2667	0.2593	0.2151	0.2076	0.2362	0.2801
Avg. (macro)	0.3242	0.3681	0.3678	0.3692	0.3577	0.3190	0.3312	**0.3771**
SimplE								
Category	0.0461	0.3591	0.3536	**0.3668**	0.0520	0.3263	0.2619	0.3367
ATC	0.3278	**0.3820**	0.3617	0.3732	0.3644	0.3410	0.3425	0.3475
Pathway	**0.7513**	0.7164	0.7299	0.7180	0.7336	0.7428	0.7448	0.7189
Interact	0.6215	0.7229	**0.7253**	0.7338	0.6488	0.6242	0.6602	0.7230
Target	0.0815	0.1128	0.1169	**0.1171**	0.0971	0.0918	0.0873	0.1163
Enzyme	0.1903	0.2442	0.2143	**0.2555**	0.2499	0.2031	0.1977	0.2304
Carrier	0.1358	**0.2544**	0.2441	0.2526	0.1881	0.1766	0.1266	0.1493
transporter	0.2242	**0.2718**	0.2189	0.2543	0.2396	0.2042	0.2417	0.2173
Avg. (macro)	0.2973	0.3829	0.3705	**0.3839**	0.3216	0.3387	0.3328	0.3549

We summarized the MRR for each relational triple and calculated the macro-averaged MRR. The highest score for each node row is shown in bold.

When we used TransE algorithm, in the *category* types, the textual models improved MRR but in other relation triple types, the MRR decreased and averaged MRR also decreased. Of the three methods that used text, the Initialization by synonyms embeddings method showed the highest macro-averaged MRR.

When we used the DistMult scoring function, the MRR decreased in *interact* and *pathway*, but on the categorical relation *category* and *ATC*, MRR was improved when we adopted the Initialization method. Initialization methods that use Name information improved the MRR of *target*, *enzyme*, *carrier* and *transporter*, which are the relations between drugs and proteins. The averaged MRR was lower than that of the models without textual information.

When we used ComplEx scoring function, The MRR decreased in the *interact* and *pathway* relation, while the MRR increased on the categorical relations and relations between drugs and proteins, these are the same tendency as the DistMult algorithm. Especially in the *category* relation, the ComplEx scoring function model without text information has a much lower MRR than TransE or DistMult-based models, but the performance was improved by using text information. The Initialization and Augmentation methods show higher macro-averaged MRR than the model without text information.

When we used SimplE scoring function, the model without text information showed the lowest macro-averaged MRR, however, the Initialization model that used the Synonyms information showed a higher MRR than the model without text information for all relation types except *pathway*, and showed the highest macro-averaged MRR in all models. These results showed that it is effective to utilize text information during updating KG embeddings under the SimplE scoring function.

These results show that the utility of textual information for learning KG embeddings depends on the scoring functions and relation types. The textual information is always useful in predicting categorical relations such as *category* and *ATC*, while the text information can be harmful for other relations and the utility depends on the scoring functions. We summarized the best setting for each relation type in Table 5. This shows there is no best single embedding method. The best method to incorporate text information including No Text and the most useful text type also depend on the relation types.

**TABLE 5:** Summary of the best settings for each relation.

	MRR	Method	Text information
Category	0.3668	SimplE	Initialization + Synonyms
ATC	0.3820	SimplE	Initialization + Name
Pathway	0.7513	SimplE	No text
Interact	0.8678	ComplEx	No text
Target	0.1243	ComplEx	Augmentation
Enzyme	0.3590	TransE	Alignment + Synonyms
Carrier	0.4155	TransE	No text
Transporter	0.3576	TransE	No text
Avg. (Macro)	0.3839	SimplE	Initialization + Synonyms

## 3.3 Discussions

### 3.1 Experimental Settings

#### 3.3.1 Analysis of the Data Imbalance of the Constructed Knowledge Graph

**FIGURE 3:**
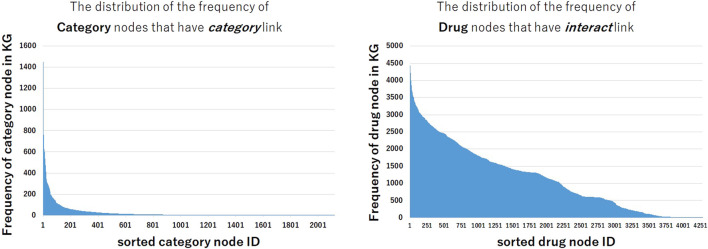
The distribution of the frequency of nodes in train data set. The frequencies of category nodes linked by *category* relation are highly imbalanced, while the frequencies of drug nodes linked by interact relation are less imbalanced.

Why some models that use text information show lower performance in *interact* and *pathway* relation and show higher performance in categorical relation and drug-protein relation? In order to analyze these tendencies, we investigated the frequency of nodes in the constructed KG. Figure 3 shows the distribution of the frequencies of category nodes that have *category* link and drug nodes that have *interact* link in train triples. Compared with the distribution of drug nodes frequency, the frequency distribution of category nodes is extremely imbalanced. The distribution shows that a small part of category nodes have the large number of triples between drugs, and many other category nodes have few triples, thus it could be difficult to predict triples that contain these nodes. Even if it is difficult to train the representation of nodes from the structural information of KG, it may be possible to predict the correct triples by utilizing the textual embeddings encoded by pre-trained BERT.

#### 3.3.2 Ablation Study of Augmentation Method

In the Augmentation method, multiple text items can be considered at the same time. Table 6 shows the results of removing each text item. Here, description and synonyms are text items that heterogeneous entities have in common, indication, pharmacodynamics, mechanism-of-action and metabolism are text items that only drug entities have, and gene-name is that only protein entities have. From the Table 6, it can be seen that the averaged MRR becomes lower regardless of which text items are removed, and these results show that all text items are effective for the link prediction task. In addition, the averaged MRR drops greatly when we exclude description or synonyms, these are the text items that many entities have. The averaged MRR also drops greatly when text information with high coverage is excluded, such as metabolism-of-action.

**TABLE 6:** Ablation study of text information on Augmentation method (ComplEx score function).

	Averaged MRR
Full text nodes	**0.3771**
- Description	0.3626
- Synonyms	0.3655
- Indication (drug)	0.3761
- Pharmacodynamics (drug)	0.3727
- Mechanism-of-action (drug)	0.3646
- Metabolism (drug)	0.3689
- Gene-name (protein)	0.3754

#### 3.3.3 Effect of Node Type Filtering

As explained in Sections 2.2.3, 3.1.4, 3.1.6, our model filters impossible negative instances by restricting the types of entities in the relations. Table 7 shows the effect of the entity type filtering. Overall, by performing entity type filtering, averaged MRR is improved. Especially, in the Augmentation method, entity type filtering is very effective, this is because the Augmentation method adds textual nodes to the graph and is more likely to create an inappropriate negative example during negative sampling.

**TABLE 7:** Comparison of averaged MRR performance for w/ and w/o entity type filtering.

	—	No text	Initialization			Alignment			Aug
—	—	—	Name	Desc	Syn	Name	Desc	Syn	—
TransE	w/ type filtering	0.3319	0.2930	0.2950	0.3015	0.3337	0.3318	0.3302	0.2883
	w/o type filtering	0.2759	0.2373	0.2265	0.2429	0.2738	0.2722	0.2753	0.1932
DistMult	w/ type filtering	0.3380	0.3206	0.2979	0.3162	0.3261	0.3274	0.3265	0.3011
	w/o type filtering	0.2217	0.2285	0.2423	0.2547	0.2462	0.2219	0.2464	0.1449
ComplEx	w/ type filtering	0.3242	0.3681	0.3678	0.3692	0.3577	0.3190	0.3312	0.3771
	w/o type filtering	0.2848	0.2906	0.2887	0.2981	0.2931	0.3052	0.3095	0.2373
SimplE	w/ type filtering	0.2973	0.3829	0.3705	0.3839	0.3216	0.3387	0.3328	0.3549
	w/o type filtering	0.2848	0.2906	0.2887	0.2981	0.2931	0.3052	0.3095	0.2373

#### 3.3.4 Analysis of text content

As can be seen from Table 5, textual information acts harmfully in some relations. In this section, we analyzed the content of the text to investigate when the text information is harmful or helpful. Table 8 shows examples of improved or worsened score ranks on the link prediction task. The examples are where the difference between the rank of textual model and the rank of non-textual model is largest, that is, examples where textual information is most useful or harmful for each relation type. In addition, we have narrowed down the cases where the better rank is 1. In example (a), the highlighted “stereoisomers” in the description of the drug entity appears in the synonyms of the category entity. Similarly, in example (b), “antiviral” in the description of the drug entity appears in the name of the ATC entity. The description of the drug entity directly mentions the category in which the drug is included, which is thought to have helped to predict the link of the categorical relation type.

**TABLE 8:** The content of the text in the examples where the difference between the rank of textual model and the rank of non-textual model is largest for each relation type. The score function SimplE was used for *category*, *ATC* and *pathway* relation and ComplEx was used for *interact* relation. The Augmentation model was selected as the model with textual information. The bold is the mention common to head and tail entities. The Description and Synonyms are partly excerpted due to space limitations.

Examples where textual information is helpful and the gap between ranks is largest for each relation type		
(a)	Relation: *category*, textual model rank:1, non-textual model rank:65	
Head	ID	DB13746 (drug entity)
	Name	*Bioallethrin*
	Desc.	*Bioallethrin refers to a mixture of two of the allethrin isomers (1R,trans;1R and 1R,trans;1S) in an approximate ratio of 1:1, where both isomers are active ingredients. A mixture of the two same* **stereoisomers** *, but in an approximate ratio of R:S in 1:3, is called esbiothrin.*
	Syn.	*Depall*é*thrine*
Tail	ID	D013237 (category entity)
	Name	*Stereoisomerism*
	Desc.	*The phenomenon whereby compounds whose molecules have the same number and kind of atoms and the same atomic arrangement, but differ in their spatial relationships.*
	Syn.	*Molecular Stereochemistry, Stereochemistry, Molecular,* **Stereoisomers** *,* **Stereoisomer**
(b)	Relation: *ATC*, textual model rank:1, non-textual model rank:25	
Head	ID	DB00369 (drug entity)
	Name	*Cidofovir*
	Desc.	*Cidofovir is an injectable* **antiviral** *medication employed in the treatment of cytomegalovirus (CMV) retinitis in patients diagnosed with AIDS.*
	Syn.	*CDV, Cidofovir anhydrous, Cidofovirum*
Tail	ID	J05A (ATC entity)
	Name	*DIRECT ACTING* **ANTIVIRALS**
	Desc.	ATC entity has no description
	Syn.	ATC entity has no synonyms
Examples where textual information is **harmful** and the gap between ranks is largest for each relation type		
(c)	Relation: *pathway*, textual model rank:25, non-textual model rank:1	
Head	ID	P51589 (protein entity)
	Name	*Cytochrome P450 2J2*
	Desc.	*This enzyme metabolizes arachidonic acid predominantly via a NADPH-dependent olefin epoxidation to all four regioisomeric cis-epoxyeicosatrienoic acids.*
	Syn.	*1.14.14.1, Arachidonic acid epoxygenase, CYPIIJ2*
Tail	ID	SMP0000695 (pathway entity)
	Name	*Etoricoxib Action Pathway*
	Desc.	*Etoricoxib (also named as Arcoxia) is a COX-2 selective inhibitor. It can be used to treat fever, pain, swelling, inflammation, and platelet aggregation.*
	Syn.	pathway entity has no synonyms
(d)	Relation: *interact*, textual model rank:4,119, non-textual model rank:1	
Head	ID	DB08893 (drug entity)
	Name	*Mirabegron*
	Desc.	*Mirabegron is a beta-3 adrenergic receptor agonist for the management of overactive bladder. It is an alternative to antimuscarinic drugs for this indication.*
	Syn.	*Mirabegron*
Tail	ID	DB00937 (drug entity)
	Name	*Diethylpropion*
	Desc.	*A appetite depressant considered to produce less central nervous system disturbance than most drugs in this therapeutic category. It is also considered to be among the safest for patients with hypertension.*
	Syn.	*alpha-Benzoyltriethylamine, alpha-Diethylaminopropiophenone, Amfepramone*

On the other hand, for the examples where the textual information is most harmful, in example (c), the description of protein “Cytochrome P450 2J2” does not directly mention the “Etoricoxib Action Pathway” pathway. In example (d), the description of each drug entity mainly describes the indication of the drug, not the relationship to other drugs. It is difficult to tell the cause of the poor rank because multiple factors may be involved, but the description of the head entity mainly explains the function and role of the head entity itself, and there is no description that mentions the relationship with the tail entity. This point is considered to be one of the causes of the textual information becoming noise.

## 4 Conclusions

We construct a new heterogeneous pharmaceutical knowledge-graph containing textual information PharmaHKG from several databases. We compared the combinations of three methods to use textual information and four scoring functions on the link prediction task. We found the utility of text information and the best combination for the link prediction depend on the target relation types. In addition, when we focus on the averaged MRR for all relation types, a method that combines SimplE and text information achieved the highest MRR, and this result showed the usefulness of text information in the link prediction task in pharmaceutical domain.

As future work, we would like to investigate a better way to incorporating text information into KG embeddings and consider other models that utilize heterogeneous graphs. We also plan to utilize the obtained representations for other tasks.

## Data Availability

Our scripts to reproduce the knowledge graph and results are available at https://github.com/tticoin/PharmaHKG-Text.

## Footnotes

1 We leave the combination of different text information in these methods for future work

## References

[B1] Abubakar A. Chedi B. Mohammed K. Haque M. (2015). Drug Interaction and its Implication in Clinical Practice and Personalized Medicine. Natl. J. Physiol. Pharm. Pharmacol. 5, 343–349. 10.5455/njppp.2015.5.2005201557

[B2] Andronis C. Sharma A. Virvilis V. Deftereos S. Persidis A. (2011). Literature Mining, Ontologies and Information Visualization for Drug Repurposing. Brief. Bioinform. 12, 357–368. 10.1093/bib/bbr005 21712342

[B3] Asada M. Miwa M. Sasaki Y. (2020). Using Drug Descriptions and Molecular Structures for Drug-Drug Interaction Extraction from Literature. Bioinformatics. 10.1093/bioinformatics/btaa907 PMC8289381 33098410

[B4] Bollacker K. Evans C. Paritosh P. Sturge T. Taylor J. (2008). “Freebase: a Collaboratively Created Graph Database for Structuring Human Knowledge,” in Proceedings of the 2008 ACM SIGMOD international conference on Management of data, Vancouver Canada, June 9–12, 2008, (New York, USA: Association for Computing Machinery), 1247–1250.

[B5] Bordes A. Usunier N. Garcia-Duran A. Weston J. Yakhnenko O. (2013). “Translating Embeddings for Modeling Multi-Relational Data,” in Neural Information Processing Systems (NIPS), 1–9.

[B6] Bordes A. Weston J. Collobert R. Bengio Y. (2011). “Learning Structured Embeddings of Knowledge Bases,” in Proceedings of the AAAI Conference on Artificial Intelligence, San Francisco, USA, August 7–11, 2011, (Palo Alto, USA: AAAI Press).

[B7] Consortium T. U. (2018). UniProt: a Worldwide Hub of Protein Knowledge. Nucleic Acids Res. 47, D506–D515. 10.1093/nar/gky1049 PMC6323992 30395287

[B8] Dechanont S. Maphanta S. Butthum B. Kongkaew C. (2014). Hospital Admissions/visits Associated with Drug-Drug Interactions: a Systematic Review and Meta-Analysis. Pharmacoepidemiol. Drug Saf. 23, 489–497. 10.1002/pds.3592 24616171

[B9] Devlin J. Chang M.-W. Lee K. Toutanova K. (2019). “BERT: Pre-training of Deep Bidirectional Transformers for Language Understanding,” in Proceedings of the 2019 Conference of the North American Chapter of the Association for Computational Linguistics: Human Language Technologies, Minneapolis USA, Jun 2–7, 2019, (Minneapolis, Minnesota: Association for Computational Linguistics), 4171–4186. 10.18653/v1/N19-1423

[B10] Doubova S. V. Reyes-Morales H. del Pilar Torres-Arreola L. Suárez-Ortega M. (2007). Potential Drug-Drug and Drug-Disease Interactions in Prescriptions for Ambulatory Patients over 50 Years of Age in Family Medicine Clinics in mexico City. BMC Health Serv. Res. 7, 1–8. 10.1186/1472-6963-7-147 17880689 PMC2080631

[B11] Gu Y. Tinn R. Cheng H. Lucas M. Usuyama N. Liu X. (2020). Domain-specific Language Model Pretraining for Biomedical Natural Language Processing. *arXiv e-prints arXiv:2007.15779*

[B12] Han X. Liu Z. Sun M. (2016). Joint Representation Learning of Text and Knowledge for Knowledge Graph Completion. *arXiv preprint arXiv:1611.04125*

[B13] Jewison T. Su Y. Disfany F. M. Liang Y. Knox C. Maciejewski A. (2014). SMPDB 2.0: Big Improvements to the Small Molecule Pathway Database. Nucleic Acids Res. 42, D478–D484. 10.1093/nar/gkt1067 24203708 PMC3965088

[B14] Ji Z. Lei Z. Shen T. Zhang J. (2020). Joint Representations of Knowledge Graphs and Textual Information via Reference Sentences. IEICE Trans. Inf. Syst. 103, 1362–1370. 10.1587/transinf.2019edp7229

[B15] Kazemi S. M. Poole D. (2018). “Simple Embedding for Link Prediction in Knowledge Graphs,” in Proceedings of the 32nd International Conference on Neural Information Processing Systems, Montréal, Canada, December 3–8, 2018, (New York, USA: Curran Associates Inc.), 4289–4300.

[B16] Kipf T. N. Welling M. (2017). “Semi-supervised Classification with Graph Convolutional Networks,” in 5th International Conference on Learning Representations, ICLR 2017, Toulon, France, April 24-26, 2017.

[B17] Lao N. Mitchell T. Cohen W. (2011). “Random Walk Inference and Learning in a Large Scale Knowledge Base,” in Proceedings of the 2011 conference on empirical methods in natural language processing, Edinburgh, UK, July 27–31, 2011, (Association for Computational Linguistics), 529–539.

[B18] Law V. Knox C. Djoumbou Y. Jewison T. Guo A. C. Liu Y. (2014). DrugBank 4.0: Shedding New Light on Drug Metabolism. Nucleic Acids Res. 42, D1091–D1097. 10.1093/nar/gkt1068 24203711 PMC3965102

[B19] Lin Y. Liu Z. Sun M. (2016). Knowledge Representation Learning with Entities, Attributes and Relations. ethnicity 1, 41–52.

[B20] Lin Y. Liu Z. Sun M. Liu Y. Zhu X. (2015). “Learning Entity and Relation Embeddings for Knowledge Graph Completion,” in Proceedings of the AAAI Conference on Artificial Intelligence, Austin, USA, January 25–30, 2015, (Palo Alto, USA: AAAI Press). 29.

[B21] Lipscomb C. E. (2000). Medical Subject Headings (MeSH). Bull. Med. Libr. Assoc. 88, 265–266. 10928714 PMC35238

[B22] Miller G. A. (1995). Wordnet: a Lexical Database for English. Commun. ACM 38, 39–41. 10.1145/219717.219748

[B23] Nickel M. Rosasco L. Poggio T. (2016). “Holographic Embeddings of Knowledge Graphs,” in Proceedings of the AAAI Conference on Artificial Intelligence, Phoenix, USA, February 12–17, 2016, (Palo Alto, USA: AAAI Press). 30.

[B24] Nickel M. Tresp V. Kriegel H.-P. (2011). ICML.A Three-Way Model for Collective Learning on Multi-Relational Data.

[B25] Paszke A. Gross S. Massa F. Lerer A. Bradbury J. Chanan G. (2019). “Pytorch: An Imperative Style, High-Performance Deep Learning Library,” in Advances in Neural Information Processing Systems 32. Editors Wallach H. Larochelle H. Beygelzimer A. d’ Alché-Buc F. Fox E. Garnett R. (New York, USA: Curran Associates, Inc.), 8024–8035.

[B26] Percha B. Altman R. B. (2013). Informatics Confronts Drug–Drug Interactions. Trends Pharmacological Sciences 34, 178–184. 10.1016/j.tips.2013.01.006 PMC3808975 23414686

[B27] Pham D.-H. Le A.-C. (2018). Learning Multiple Layers of Knowledge Representation for Aspect Based Sentiment Analysis. Data Knowledge Eng. 114, 26–39. 10.1016/j.datak.2017.06.001

[B28] Riechelmann R. P. Tannock I. F. Wang L. Saad E. D. Taback N. A. Krzyzanowska M. K. (2007). Potential Drug Interactions and Duplicate Prescriptions Among Cancer Patients. J. Natl. Cancer Inst. 99, 592–600. 10.1093/jnci/djk130 17440160

[B29] Schlichtkrull M. Kipf T. N. Bloem P. Van Den Berg R. Titov I. Welling M. (2018). Modeling Relational Data with Graph Convolutional Networks. in European Semantic Web Conference. Springer, 593–607. 10.1007/978-3-319-93417-4_38

[B30] Schriml L. M. Arze C. Nadendla S. Chang Y.-W. W. Mazaitis M. Felix V. (2012). Disease Ontology: a Backbone for Disease Semantic Integration. Nucleic Acids Res. 40, D940–D946. 10.1093/nar/gkr972 22080554 PMC3245088

[B31] Sheikhalishahi S. Miotto R. Dudley J. T. Lavelli A. Rinaldi F. Osmani V. (2019). Natural Language Processing of Clinical Notes on Chronic Diseases: Systematic Review. JMIR Med. Inform. 7, e12239. 10.2196/12239 31066697 PMC6528438

[B32] Simon C. Davidsen K. Hansen C. Seymour E. Barnkob M. B. Olsen L. R. (2019). Bioreader: a Text Mining Tool for Performing Classification of Biomedical Literature. BMC bioinformatics 19, 165–170. 10.1186/s12859-019-2607-x PMC7394276 30717659

[B33] Socher R. Chen D. Manning C. D. Ng A. (2013). Reasoning with Neural Tensor Networks for Knowledge Base Completion. Adv. Neural Inf. Process. Syst. 26, 926–934.

[B34] Suchanek F. M. Kasneci G. Weikum G. (2007). “Yago: a Core of Semantic Knowledge,” in Proceedings of the 16th international conference on World Wide Web, Alberta Canada, May 8–12, 2007, (New York, USA: Association for Computing Machinery), 697–706.

[B35] Toutanova K. Chen D. Pantel P. Poon H. Choudhury P. Gamon M. (2015). “Representing Text for Joint Embedding of Text and Knowledge Bases,” in Proceedings of the 2015 conference on empirical methods in natural language processing, Lisbon, Portugal, September 17–21, 2015, (Association for Computational Linguistics), 1499–1509.

[B36] Trouillon T. Welbl J. Riedel S. Gaussier É. Bouchard G. (2016). “Complex Embeddings for Simple Link Prediction,” in International Conference on Machine Learning, New York, USA, June 19–24, 2016, (PMLR), 2071–2080.

[B37] Wang X. Ji H. Shi C. Wang B. Ye Y. Cui P. (2019). “Heterogeneous Graph Attention Network,” in The World Wide Web Conference, San Francisco, USA, May 13–17, 2019, (New York, USA: Association for Computing Machinery), 2022–2032.

[B38] Wang X. Zhang S. Wu Y. Yang X. (2021). Revealing Potential Drug-Disease-Gene Association Patterns for Precision Medicine. Scientometrics, 1–26.

[B39] Wang Y. Zhang H. Shi G. Liu Z. Zhou Q. (2020). A Model of Text-Enhanced Knowledge Graph Representation Learning with Mutual Attention. IEEE Access 8, 52895–52905. 10.1109/access.2020.2981212

[B40] Wishart D. S. Feunang Y. D. Guo A. C. Lo E. J. Marcu A. Grant J. R. (2017). DrugBank 5.0: a Major Update to the DrugBank Database for 2018. Nucleic Acids Res. 46, D1074–D1082. 10.1093/nar/gkx1037 PMC5753335 29126136

[B41] Wolf T. Debut L. Sanh V. Chaumond J. Delangue C. Moi A. (2020). “Transformers: State-Of-The-Art Natural Language Processing,” in Proceedings of the 2020 Conference on Empirical Methods in Natural Language Processing: System Demonstrations, November 16–20, 2020, (Online: Association for Computational Linguistics), 38–45.

[B42] Wu H. Xing Y. Ge W. Liu X. Zou J. Zhou C. (2020). Drug-drug Interaction Extraction via Hybrid Neural Networks on Biomedical Literature. J. Biomed. Inform. 106, 103432. 10.1016/j.jbi.2020.103432 32335223

[B43] Xie R. Liu Z. Sun M. (2016). “Representation Learning of Knowledge Graphs with Hierarchical Types,” in IJCAI, 2965–2971.

[B44] Yang B. Yih W.-t. He X. Gao J. Deng L. (2014). Embedding Entities and Relations for Learning and Inference in Knowledge Bases.

[B45] Zeng D. Liu K. Lai S. Zhou G. Zhao J. (2014). “Relation Classification via Convolutional Deep Neural Network,” in Proceedings of COLING 2014, the 25th international conference on computational linguistics: technical papers, Dublin, Ireland, August 23–29, 2014, (Dublin City University and Association for Computational Linguistics), 2335–2344.

[B46] Zhao Z. Yang Z. Luo L. Lin H. Wang J. (2016). Drug Drug Interaction Extraction from Biomedical Literature Using Syntax Convolutional Neural Network. Bioinformatics 32, 3444–3453. 10.1093/bioinformatics/btw486 27466626 PMC5181565

[B47] Zheng D. Song X. Ma C. Tan Z. Ye Z. Dong J. (2020). “DGL-KE: Training Knowledge Graph Embeddings at Scale,” in Proceedings of the 43rd International ACM SIGIR Conference on Research and Development in Information Retrieval, Virtual Event China, July 25–30, 2020, (New York, NY, USA: Association for Computing MachinerySIGIR), 739–748.

